# Surveillance Strategy in Duck Flocks Vaccinated against Highly Pathogenic Avian Influenza Virus

**DOI:** 10.3201/eid3101.241140

**Published:** 2025-01

**Authors:** Sophie Planchand, Timothée Vergne, Jean-Luc Guérin, Séverine Rautureau, Guillaume Gerbier, Sébastien Lambert

**Affiliations:** National Veterinary School of Toulouse, Toulouse, France (S. Planchand, T. Vergne, J.-L. Guérin, S. Lambert); French Ministry of Agriculture, Food Sovereignty and Forestry, Paris, France (S. Rautureau, G. Gerbier)

**Keywords:** Highly pathogenic avian influenza virus, influenza, zoonoses, ducks, vaccination, surveillance, mathematical modeling, poultry, France

## Abstract

Since 2016, epizootics of highly pathogenic avian influenza (HPAI) virus have threatened the poultry sector in Europe. Because conventional prevention and control measures alone were insufficient in some contexts, the European Commission authorized poultry vaccination in 2023. Subsequently, France launched a nationwide duck vaccination campaign combined with a comprehensive surveillance plan. We used a mathematical model to simulate the transmission of HPAI viruses in vaccinated duck flocks and assess the effectiveness of a wide range of surveillance strategies. Sampling and testing dead ducks every week (enhanced passive surveillance) was the most sensitive (≈90%) and the most timely strategy. Active surveillance through monthly testing of a cross-sectional sample of live ducks was the least sensitive and timely strategy. Thus, we advise focusing HPAI surveillance efforts on enhanced passive surveillance and reducing active surveillance of live ducks.

During 2000–2016, one quarter of major animal disease outbreaks worldwide were caused by highly pathogenic avian influenza (HPAI) viruses (*1*). Since then, the emergence of HPAI clade 2.3.4.4b virus in 2016 has caused major epizootics at an accelerating pace across several continents (*2*). Those RNA viruses mainly infect birds and represent a substantial threat to the poultry sector. In poultry, HPAI causes direct losses because of high illness rates and a high case-fatality risk of up to 100% (*3*). HPAI also is responsible for indirect economic costs related to outbreak prevention and management and market losses (*4*). Increasing transmissions to mammals also have been observed throughout the world (*5*,*6*). Although the number of human cases of infection remains limited (*7*), HPAI viruses must be carefully managed to reduce spillover events into mammal species and limit their zoonotic potential.

Since 2016, HPAI epizootics have wreaked havoc in the poultry sector. During previous epizootics in Europe, France was most impacted of all countries. The 2021–2022 HPAI epizootic was the most devastating, and almost 1,400 outbreaks were reported from poultry farms in France (*8*). The unprecedented scale of those epizootics and nearly annual recurrences, showed that the conventional prevention and control strategies predominantly aimed at biosecurity were no longer sufficient to control HPAI. Vaccination, which was previously prohibited in the European Union (EU) to ease trade between Member States, was then reconsidered (*9*), and the EU finally authorized vaccination in February 2023 (*10*). Then, in May 2023, vaccination was recognized as a valuable flanking option by the World Organisation for Animal Health (*11*).

In October 2023, France launched a nationwide preventive vaccination campaign, targeting all ducks raised and intended for human consumption (*12*). France decided to only vaccinate ducks because of their prominent epidemiologic role in HPAI transmission (*12*). Duck farms are associated with the highest risk for viral spread because of the high receptivity and infectivity of ducks (*13**–**15*) and the outdoor grazing system used for ducks producing foie gras (*16*). Because HPAI epizootics in France have been mostly driven by a few primary introductions followed by between-farm spread (*17*), the vaccination campaign in France aimed to reduce viral spread by both limiting the susceptibility of uninfected ducks and the viral excretion and infectivity of vaccinated infected ducks.

Silent circulation is one of the main risks associated with HPAI vaccination, because vaccination drastically reduces illness and case-fatality risk (*18*). Consequently, vaccination must be organized in conjunction with strict monitoring protocols in vaccinated flocks. In line with the EU Delegated Regulation No. 2023/361 (*10*), France implemented a comprehensive compulsory surveillance program on vaccinated flocks (*19*). The purpose of that program is to detect HPAI in vaccinated flocks with high probability and as early as possible to convince international trade partners that the virus in vaccinated populations is under control. Using European Food Safety Agency (EFSA) terminology (*20*), surveillance can be passive, enhanced passive, or active. Passive and enhanced passive surveillance protocols have 2 stages. In the first stage of passive surveillance, infection is suspected when HPAI clinical or paraclinical signs are observed. In the first stage of enhanced passive surveillance, the farmer or technician takes weekly tracheal or oropharyngeal swab samples from all dead ducks with a maximum of 5 ducks per vaccinated holding; samples are then tested by reverse transcription PCR (RT-PCR) in a recognized laboratory (*21*). If HPAI is suspected during passive surveillance or a positive HPAI virus sample is collected during enhanced passive surveillance, a second stage consists of an official veterinarian taking tracheal or oropharyngeal swab samples that are tested in a certified laboratory (*12*,*21*). Last, active surveillance consists of a single stage in which an official veterinarian takes cross-sectional tracheal or oropharyngeal swab samples from 60 live ducks at least every 30 days for RT-PCR testing (*12*).

A recent EFSA report quantified the effectiveness of different surveillance strategies at a multifarm level, including preventively vaccinated flocks, using scenario tree models (*20*). We adopted a different approach, using mechanistic modeling at the farm level to quantify the effectiveness of a wide range of surveillance protocols in vaccinated flocks. 

## Methods

### Modeling HPAI Virus Transmission within Vaccinated Flocks

First, we simulated HPAI virus transmission in a typical vaccinated duck flock in France. We considered a flock of 6,400 mule ducks (a hybrid of *Anas platyrhynchos domesticus* × *Cairina moschata domestica*) raised for foie gras production (*22*). We focused on the first stage of production, the breeding stage, which lasts for 84 days, because the second (fattening) stage only lasts 12 days and is usually performed in different farms than the first stage. We assumed that ducks received a first vaccine dose of VOLVAC B.E.S.T AI + ND KV, emulsion for injection (Boehringer Ingleheim, https://www.boehringer-ingelheim.com) at 10 days of age, then a second dose 18 days later, as recommended when the preventive vaccination campaign began (*23*).

We used a stochastic SEIRD (susceptible, exposed, infectious, recovered, or dead) model to simulate within-flock transmission of HPAI viruses. In brief, the model assumed that ducks could be categorized into mutually exclusive compartments according to their status, namely: susceptible (S), exposed (E; i.e., infected but not yet infectious), infectious (I), recovered (R), or dead (D). At the start of the simulations, we considered all ducks to be susceptible. For each simulation, we used a random date between the day of the first vaccination dose (day 10) and the last day of the production cycle (day 84) to simulate the introduction of an HPAI virus. We modeled the virus introduction by moving 1 random bird from the S compartment into the E compartment. After a certain latent period, the duck entered the I compartment and was then able to infect susceptible ducks. At the end of its infectious period, the duck could either recover or die from the infection.

We assumed that all ducks received the vaccine (i.e., vaccination coverage in the flock was 100%). However, because some vaccinated ducks might not develop protective immunity, we tested various scenarios in which 70%, 80%, or 90% of ducks in the vaccinated flock were immune to represent effective vaccination coverage. We considered that the population was composed of 2 subpopulations and that immune and nonimmune ducks could mix freely. We assumed immune ducks had developed protective immunity and were therefore associated with different parameters (Appendix). 

### Model Calibration

The model had 11 parameters: the day of virus introduction on the farm, the transmission rate, the natural mortality rate, the case-fatality risks for immune and nonimmune ducks, the average durations of the latent and infectious periods for immune and nonimmune ducks, and the relative reductions in susceptibility (protective immunity) and in infectivity (reduction in viral shedding) for immune ducks (Appendix Table). For the immune population, we assumed no vaccine-induced protective immunity before the second vaccine dose at 28 days of age (preimmunity phase). Then, we assumed immunity gradually built between 28 and 35 days of age (transition phase) and was fully reached at 35 days of age (immunity phase) until the end of the production cycle (*24*). We therefore considered that the average duration of the infectious period, the case-fatality rate, and the susceptibility and infectivity of immune ducks decreased linearly during the transition phase, moving from the value associated with unvaccinated ducks to that of immune ducks.

Our model assumed a 95% reduction in case-fatality risk for immune ducks compared with unvaccinated ones and a baseline scenario with no reduction for nonimmune ducks. However, vaccination might still reduce case-fatality risk in nonimmune ducks (*25*), which could affect the effectiveness of surveillance strategies that are based on mortality. Therefore, we conducted a sensitivity analysis using 3 assumptions: 0%, 50%, and 95% reduction in case-fatality risk compared with unvaccinated ducks.

### Quantification of HPAI Virus Transmission within Vaccinated Flocks

We assessed the impact of vaccination on HPAI virus transmission by comparing outbreak characteristics in vaccinated and unvaccinated flocks. We defined an outbreak as a simulation where >5 ducks became infected after the first infected duck. Directly from the simulations, we calculated the probability of outbreak occurrence and the proportion of ducks that became infectious within 14 days after the virus introduction. Using the next-generation matrix method, we also computed between-bird reproduction number (R) in each of those scenarios (*26*). For the scenarios involving vaccinated flocks, we also assessed the effect of the immune status of the first infected duck on the simulation outputs. Finally, we defined different scenarios of the time of virus introduction to investigate how the immunity-building period influenced the effect of vaccination on HPAI virus transmission. To do so, we simulated virus introduction during the preimmunity, transition, and immunity phases.

For unvaccinated flocks, we ran a total of 1,500 simulations, dedicating 500 simulations to a virus introduction in each of the 3 phases. For vaccinated flocks, we ran a total of 4,500 simulations: 1,500 simulations for each of the 3 phases in which we introduced the virus and 500 simulations of which were dedicated to each status of the first infected duck (i.e., nonimmune, immune, or randomly selected in the population). When we randomly selected the first infected duck, we assumed a probability of 0.1 to be nonimmune and probability of 0.9 to be immune for an effective vaccination coverage of 90%.

### Effectiveness of Surveillance Strategies

After we simulated infection, we integrated different surveillance strategies into the model to quantify surveillance strategy performance for detecting HPAI viruses at the farm level. We defined 5 surveillance strategies, which were inspired by those described in regulations in France and Europe.

#### Surveillance Strategy Definitions

The strategies we defined included 3 passive surveillance strategies (P1, P2, and P3) based on daily or weekly duck mortality thresholds, an enhanced passive surveillance strategy based on the regular testing of dead ducks, and an active surveillance strategy based on regular testing of a cross-sectional sample of 60 live ducks. We further refined those 5 strategies by using different mortality thresholds to trigger an alert in P1, P2, and P3 (Table 1); varying the sample size (3, 5, or 7 dead ducks) and the sampling frequency (7 or 14 days) during enhanced passive surveillance; and varying sampling frequency (20, 30, or 40 days) for active surveillance. For active surveillance, we also added an extra sampling session on the last day of the production cycle (Table 2). For enhanced passive and active surveillance strategies, we assumed that the probability of obtaining a positive RT-PCR test result was 1 for ducks that died because of the infection and for live infectious ducks (i.e., perfect test sensitivity).

**Table 1:** Description of surveillance strategies used in duck flocks vaccinated against highly pathogenic avian influenza virus*

Surveillance strategy	Description	Case definition assumed to trigger an alert	Additional information
Passive			
P1	Daily proportion of dead ducks found on the farm	Daily percentage of dead ducks exceeds a predefined threshold	Thresholds used: 0.1%, 0.2%, and 0.5%
P2	Weekly proportion of dead ducks found on the farm	Weekly percentage of dead ducks exceeds a predefined threshold	Thresholds used: 0.5%, 1%, and 3%
P3	Ratio of the daily influenza mortality to the natural daily mortality	Ratio exceeds a predefined threshold	Thresholds used: 5×, 10×, and 20× natural daily mortality
Enhanced passive	RT-PCR testing of a predefined number of randomly selected dead ducks or of all dead ducks if less than the predefined number, at predefined intervals	At least 1 selected dead duck tested positive	Intervals used: 7 and 14 d; no. selected dead ducks: 3, 5, and 7
Active	RT-PCR testing of 60 live ducks randomly sampled at predefined intervals	At least 1 selected live duck tested positive	Intervals used: 20, 30, and 40 d


*RT-PCR, reverse transcription PCR.

**Table 2:** Summary of the surveillance events over the entire production cycle in a study of surveillance strategies in duck flocks vaccinated against highly pathogenic avian influenza virus*

Events	Days in production cycle																	
	10	18	20	25	28	30	32	35	39	40	46	53	60	67	74	80	81	84
Vaccine doses	X				X													
% Immunity†	0	0	0	0	In	In	In	In	100	100	100	100	100	100	100	100	100	100
Surveillance events																		
Passive‡	D	D	D	D	D	D	D	D	D	D	D	D	D	D	D	D	D	D
Enhanced passive																		
Every 7 d		X		X			X		X		X	X	X	X	X		X	
Every 14 d				X					X			X		X			X	
Active																		
Every 20 d			X							X			X			X		X
Every 30 d						X							X					X
Every 40 d										X						X		X

*D, daily; In, increasing gradually; P, passive surveillance. †Refers to immunity in vaccinated and immune ducks (70%, 80%, or 90% of the flock immune). ‡Passive surveillance has 3 strategies: P1, daily proportion of dead ducks found on the farm; P2, weekly proportion of dead ducks found on the farm; and P3, ratio of the daily mortality to the natural daily mortality.

#### Assessing the Effectiveness of the Surveillance Strategies

We compared surveillance strategies on the basis of sensitivity (proportion of outbreaks that triggered an alert) and timeliness (number of days between virus introduction and the alert). We considered that an alert was triggered when mortality thresholds were exceeded in the passive surveillance strategies or when >1 infected duck (dead or alive) was sampled in the first stage of the enhanced passive and active strategies (Table 1). We assumed that the second stage (official sampling) of passive and enhanced passive strategies would confirm the infection and that sensitivity would therefore remain the same.

We ran simulations until we obtained 5,000 outbreaks (i.e., simulations in which >5 ducks became infected after the first infected duck). We randomly introduced the virus during the transition and immunity phases because we wanted to compare the effectiveness of surveillance strategies in flocks with partially or fully immune ducks.

To examine situations in which silent spread could occur, we further characterized outbreaks that were never detected. We recorded the outbreak size (cumulative number of infected and dead ducks), outbreak duration (number of days with infected ducks on the farm), and the day of virus introduction. Finally, we calculated the proportion of undetected outbreaks in which infected ducks still existed at the end of the production cycle.

## Results

### Quantification of HPAI Virus Transmission within Vaccinated Flocks

**Figure 1:**
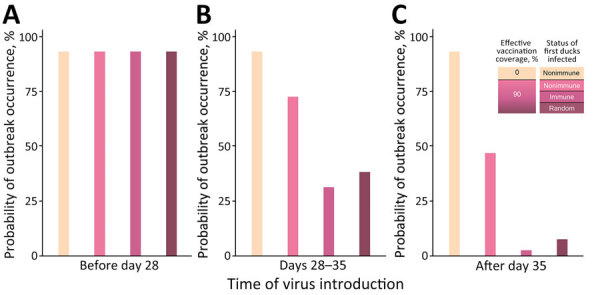
Probability of outbreak occurrence in nonvaccinated and preventively vaccinated duck flocks in a study of surveillance strategies in duck flocks vaccinated against highly pathogenic avian influenza virus. Graphs show different timings of virus introduction: A) preimmunity phase, in which the virus was introduced into the flock when ducks were not yet immune (i.e., before day 28); B) transition phase, in which the virus was introduced between day 28 and day 35; and C) immunity phase, in which the virus was introduced once immunity was fully reached. Each probability was calculated based on 500 stochastic simulations of the model. Effective vaccination coverage in vaccinated flocks was assumed to be 90%. Outbreak was defined as a simulation where >5 ducks became infected after the first infected duck. When we randomly selected the status of the first infected duck, we assumed a probability of 0.1 to be nonimmune and 0.9 to be immune.

Without vaccination, we estimated the probability of outbreak occurrence (i.e., the probability of having >5 infections after the first infected duck) at 93% (Figure 1). When effective vaccination coverage was 90%, the probability decreased to 38% when the virus was introduced during the transition phase (between 28 and 35 days) and to 8% when the virus was introduced during the immunity phase (after 35 days). The probability of outbreak occurrence also strongly depended on the status of the first infected duck. In the immunity phase, if the first infected duck was not immune, we estimated the probability at 47%, but probability decreased to 3% if the first infected duck was immune. We obtained equivalent trends when assuming effective vaccination coverage of 70% and 80% (Appendix Figures 1, 2).

In the simulated outbreaks with no vaccination, a median of 99% of ducks (95% prediction interval [95% PI] 97%­–100%) became infectious within 14 days after virus introduction. When the virus was introduced in the immunity phase of a flock with an effective vaccination coverage of 90%, a median of 0.3% (95% PI 0.078%–2.5%) of ducks became infectious within 14 days after virus introduction. Finally, we estimated the median R to be 16 (95% PI 7.6–40) without vaccination and 1.7 (95% PI 0.8–3.9) for an effective vaccination coverage of 90% and a virus introduction in the immunity phase.

### Effectiveness of Different Surveillance Strategies

**Figure 2:**
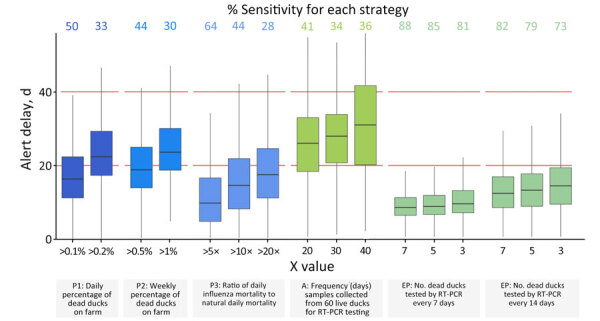
Comparison of the sensitivity and alert delay of different surveillance strategies in duck flocks vaccinated against highly pathogenic avian influenza virus. Effective vaccination coverage in vaccinated flocks was assumed to be 90%. For each of the surveillance strategies, 2 or 3 scenarios were tested by varying the value of X (Table 1). For passive surveillance strategies P1, P2, and P3, X referred to mortality thresholds (Table 1). For active surveillance, X referred to the frequency with which samples were taken from 60 live ducks on the farm. For enhanced passive surveillance, X referred to the number of dead ducks sampled each time. For each of these scenarios, the sensitivity and alert delay were compared. Sensitivity was the percentage of outbreaks out of 5,000 that triggered an alert. Alert delay was the distribution of the number of days between the virus introduction and the alert, out of 5,000 outbreaks. Red horizontal lines indicate upper and lower limits for alert delay. Horizontal lines within boxes indicate medians, box top and bottom edges indicate 50% prediction intervals, and whiskers indicate ranges. Percent sensitivity is shown above plots. A, active surveillance; EP, enhanced passive surveillance; P, passive surveillance; RT-PCR, reverse transcription PCR.

We found enhanced passive surveillance strategies were the most sensitive strategies, assuming an effective vaccination coverage of 90% and a virus introduction during the transition or immunity phase. Among the outbreaks, 81% were detected with a weekly sampling of 3 dead ducks, 85% with a sampling of 5 dead ducks, and 88% with a sampling of 7 dead ducks (Figure 2). We found the biweekly version of that strategy was also highly sensitive and had sensitivities up to 82%. For the passive surveillance strategies P1 and P3, which were based on daily mortality, sensitivity ranged from 28%–64%, depending on the thresholds. The P2 (weekly mortality) and active (live bird sampling) strategies were the least sensitive. Even if we considered P2 at a 0.5% threshold, or active surveillance with a 20-day sampling frequency, the sensitivity did not exceed 50% (Figure 2).

The enhanced passive surveillance strategy had the shortest alert delay, irrespective of the sampling frequency, closely followed by P3 (Figure 2). The weekly testing of 5 dead ducks had a median alert delay of 9 (95% PI 2.5–20) days, and the biweekly testing of 5 dead ducks had a median alert delay of around 14 (95% PI 4.2–29) days. In contrast, running RT-PCR tests on 60 randomly sampled live ducks every 30 days (active strategy) had a median alert delay of 28 (95% PI 7.6–53) days.

Of 5,000 simulated outbreaks in vaccinated flocks with an effective vaccination coverage of 90%, only 7% were not detected by any of the surveillance strategies. In those outbreaks, the median number of infectious ducks was 8 (95% PI 5–35), the median number of infection-related deaths was 1 (95% PI 0–5), and the median duration was 8 (95% PI 3–15) days. Among those outbreaks, 20% no longer had infectious ducks at the end of the production cycle. For the other 80%, we observed a low prevalence on the last day (mean within-flock prevalence was 0.3%) and the median day of virus introduction was day 77 (95% PI day 69–82), close to the end of the production cycle of day 84.

When the level of effective vaccination coverage decreased from 90% to 80% and 70%, all alert delays decreased, and sensitivity increased. As expected, the sensitivity estimates of the passive surveillance strategies (P1, P2, P3) increased substantially, but the enhanced passive strategies nonetheless remained the most sensitive and timely (Appendix Figures 4, 5). Similarly, when we considered that nonimmune ducks had an intermediate or reduced case-fatality rate, the enhanced passive strategies remained the most sensitive and timely. However, we noticed that the more nonimmune ducks survived the infection, the more the sensitivity of passive and enhanced passive strategies decreased (Appendix Figures 3–5).

## Discussion

Vaccination of domestic poultry flocks against HPAI viruses is a promising control tool to complement existing measures (*15*). During October 2023–September 2024, the virus was detected in only 13 poultry farms in France, only 2 of which were vaccinated duck farms (*27*). Although a lower level of virus circulation was observed in Europe overall in 2023–2024, a recent study suggested that vaccination reduced the expected epizootic size by 92%–98% (C. Guinat et al., unpub. data, https://doi.org/10.1101/2024.08.28.609837).

However, because illness and deaths are strongly reduced in immune ducks (*28*), passive HPAI surveillance becomes much less effective in detecting the presence of the virus in vaccinated flocks (*18*). Therefore, vaccination use must be combined with effective surveillance strategies. In this study, we developed a mathematical model to compare the effectiveness of different HPAI surveillance strategies in preventively vaccinated mule duck flocks.

In vaccinated flocks with a virus introduction during the immunity phase, we observed a 10-fold reduction in the number of outbreaks and a 100-fold reduction in the number of ducks that became infectious within 14 days after virus introduction compared with unvaccinated flocks. Those reductions were expected because we assumed, based on results from experimental studies, that immune ducks were 90% less susceptible and that the amount of virus shedding was reduced by 90% and duration of virus shedding was reduced by 81% (*29*). Experimental studies under field conditions would enable comparisons of the results from our model by quantifying the within-flock virus transmission.

Our results suggest that performing virologic tests on dead ducks (i.e., enhanced passive surveillance) was the most sensitive strategy and had the shortest time delay of detection, regardless of our assumptions on the effective vaccination coverage or the case-fatality rate (Appendix Figures 3–5). We focused only on the first stage of enhanced passive surveillance strategies. In case of positive RT-PCR results, the flock would be resampled by an official veterinarian. In our model, we assumed that sensitivity would remain the same after the second sample. However, given our results, we advise that the second sampling would also be performed on dead ducks because confirming HPAI virus in a sample of live birds might lead to a lower sensitivity. EFSA also recommended performing virologic tests on dead ducks to prove flocks are free of disease and to get high early detection sensitivity in an area with preventively vaccinated flock (*20*). Despite using a different method, we reached the same conclusion, which provides additional evidence in support of the enhance passive surveillance strategy.

None of the surveillance strategies showed 100% sensitivity, and 7% of outbreaks were not detected by any of the surveillance strategies in our model. As expected, those outbreaks were hard to detect because of very low within-flock prevalences and short outbreak duration. Nonetheless, 80% of those outbreaks still had infected ducks at the end of the production cycle that were not detected, even by the active surveillance performed on the last day of production. However, those simulated outbreaks that still had infected ducks at the end of the production cycle were associated with a late introduction of the virus and a very low prevalence on the last day. Higher prevalence could be expected if, contrary to our model assumption, protective immunity did not last until the end of the production cycle. However, in that case, late outbreaks would be easier to detect, thus mitigating the risk for virus spread. Developing a between-farm transmission model could quantify the risk represented by undetected outbreaks to other farms.

One limitation of our study is that we did not assess alternative sampling strategies, such as environmental sampling (i.e., molecular testing performed on dust or aerosol samples), which might be valuable and warrant further assessment in vaccinated flocks (*20*). In addition, we focused only on RT-PCR, even though other diagnostic methods, such as rapid antigen assays, exist. Those methods would be relevant in field conditions because they enable rapid results without special laboratory equipment. However, the sensitivity of those assays has not been assessed in field conditions and is assumed to be low compared with RT-PCR (*20*).

In conclusion, surveillance of flocks vaccinated against HPAI virus is a serious challenge. Our modeling results suggest that virologic tests on dead birds, conducted either once a week or every 2 weeks, is a promising strategy, but that virologic tests on samples from live birds are less effective. Passive surveillance is also useful, especially when the level of immunity is not very high or when vaccination fails. For example, passive surveillance detected the only 2 outbreaks that occurred in vaccinated duck flocks in France in 2024 (*27*). Future studies could evaluate combined strategies instead of comparing strategies in isolation, and additional criteria, such as cost, workload, and bird stress, could also be evaluated to refine the overall strategy. In the meantime, we advise focusing HPAI surveillance efforts on enhanced passive surveillance and reducing active surveillance of live ducks.

Appendix Additional information on surveillance strategies in duck flocks vaccinated against highly pathogenic avian influenza virus.
