# Supplementary material for: Surveillance Strategy in Duck Flocks Vaccinated against Highly Pathogenic Avian Influenza Virus
Source: Emerg Infect Dis. 2025 Jan;31(1):115–22. doi: 10.3201/eid3101.241140 (PMC11682810; doi:10.3201/eid3101.241140)
Supplement: Appendix — Additional information on surveillance strategies in duck flocks vaccinated against highly pathogenic avian influenza virus. [file 24-1140-Techapp-s1.pdf]

Article DOI: https://doi.org/10.3201/eid3101.241140

EID cannot ensure accessibility for supplementary materials supplied by authors. Readers who have difficulty accessing supplementary content should contact the authors for assistance.

Surveillance Strategy in Duck Flocks Vaccinated against Highly Pathogenic Avian Influenza Virus

Appendix

Force of Infection Equations

The forces of infection exerted on susceptible nonimmune ducks are given by equations (1) and on immune ducks by equation (2) below:

$$\lambda_{ni}(t) = \beta \frac{I_{ni}(t)}{N(t)} + f\beta \frac{\mathrm{I}_i(t)}{N(t)} \quad (1)$$

$$\lambda_{i}(t) = g\left(\beta \frac{I_{ni}(t)}{N(t)} + f\beta \frac{\mathrm{I}_i(t)}{N(t)}\right) \quad (2)$$

where β is the transmission rate between nonimmune ducks, $I_{ni}(t)$, $I_i(t)$, and $N(t)$ are the number of infectious nonimmune ducks at time t, the number of infectious immune ducks at time t and the total number of ducks at time t, respectively, and f and g are the relative infectivity and susceptibility of immune ducks (as compared to nonimmune ducks), respectively.

Appendix Table. Model parameter values or distributions used to model highly pathogenic avian influenza transmission within a vaccinated flock with both immune and nonimmune ducks

Parameters	Description *(unit)*	Assumed value or distribution	Reference
t_0	Day of virus introduction	Uniform(min = 10, max = 84)	NA
β	Transmission rate (day^{-1})	Gamma(rate = 3.1; shape = 11.1)<br>Mean = 3.6; Variance = 1.2	Derived from (*1*)
r_M	Natural mortality rate	Gamma(rate = 6613.8; shape = 1.2)<br>Mean = 1.8 × 10^{-4}; Variance = 2.7 × 10^{-8}	Derived from (*1*)
Unvaccinated ducks			
$case_F$	Probability of dying of the disease	Gamma(rate = 228.3; shape = 157.8)<br>Mean = 0.69; Variance = 3 × 10^{-3}	Derived from (*1*)
Vaccinated but nonimmune duck			
$\mu_{E_{ni}}$	Mean duration of the latent period (day)	Gamma(rate = 6.2; shape = 2.9)<br>Mean = 0.47; Variance = 0.076	Derived from (*1*)
$\mu_{I_{ni}}$	Mean duration of the infectious period (day)	Gamma(rate = 2.7; shape = 13.6)<br>Mean = 5; Variance = 1.8	Derived from (*1*)
$case_{F_{ni}}$	Probability of dying of the disease	$k_{case_{F_{ni}}} \times case_F$	NA

Parameters	Description *(unit)*	Assumed value or distribution	Reference
$k_{case_{F_{ni}}}$	Reduction factor for the probability of dying of the disease	1, 0.5, and 0.05	NA
Vaccinated immune duck			
g	Relative susceptibility	0.1	Assumed in accordance with (*2*)
μ_{E_i}	Mean duration of the latent period (day)	$\mu_{E_{ni}}$	(*2*)
μ_{I_i}	Mean duration of the infectious period (day)	k_{μ_I} * $\mu_{I_{ni}}$	NA
k_{μ_I}	Reduction factor for the mean duration of the infectious period	0.19	(*2*)
f	Relative infectivity	0.1	Assumed in accordance with (*2*)
$case_{F_i}$	Probability of dying of the disease	$k_{case_{F_i}} \times case_F$	NA
$k_{case_{F_i}}$	Reduction factor for the probability of dying of the disease	0.05	Assumed in accordance with (*3*,*4*)

NA, not applicable

References

1. Vergne T, Gubbins S, Guinat C, Bauzile B, Delpont M, Chakraborty D, et al. Inferring within-flock transmission dynamics of highly pathogenic avian influenza H5N8 virus in France, 2020. Transbound Emerg Dis. 2021;68:3151–5. PubMed https://doi.org/10.1111/tbed.14202

2. Grasland B, Schmitz A, Niqueux E, Busson R, Morin N, Guillemoto C, et al. Experimental vaccination of mulard ducks in breeding against a highly pathogenic avian influenza virus A(H5N1) clade 2.3.4.4b. Interim report: Experimental evaluation of the transmission of an HPAI virus A(H5N1) clade 2.3.4.4b within a population of mulard ducks vaccinated and challenged at 7 weeks of age [in French]. Lyon: Agence Nationale de Sécurité Sanitaire; 2023.

3. Hsu SM, Chen THH, Wang CH. Efficacy of avian influenza vaccine in poultry: a meta-analysis. Avian Dis. 2010;54:1197–209. PubMed https://doi.org/10.1637/9305-031710-Reg.1

4. Tatár-Kis T, Dán Á, Felföldi B, Bálint Á, Rónai Z, Dauphin G, et al. Virus-like particle based vaccine provides high level of protection against homologous H5N8 HPAIV challenge in mule and Pekin duck, including prevention of transmission. Avian Dis. 2018;63:193–202. PubMed https://doi.org/10.1637/11882-042718-Reg.1

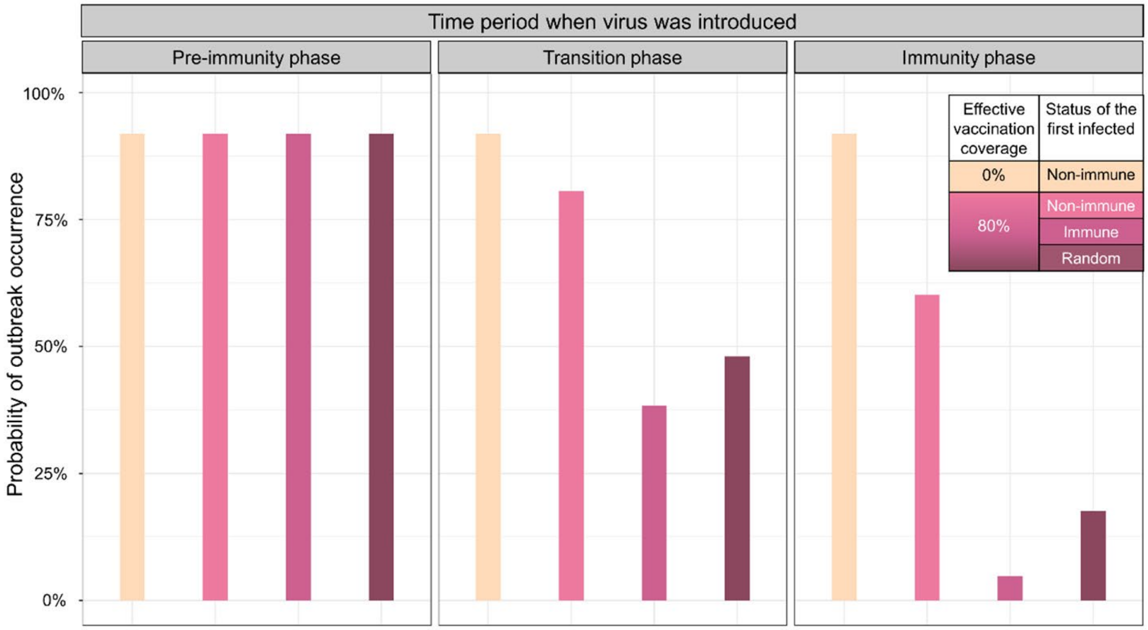

Appendix Figure 1. Probability of outbreak occurrence in unvaccinated (beige bars) and preventively vaccinated duck flocks (pink bars), for different timings of virus introduction. Effective vaccination coverage in vaccinated flocks was assumed to be 80%. Outbreak was defined as a simulation where at least five ducks became infected following the first infected duck. Left panel: the virus was introduced into the flock when ducks were not yet immune (i.e., before day 28); middle panel: the virus was introduced during the transition phase (i.e., between day 28 and day 35); right panel: the virus was introduced once immunity was fully reached (i.e., after day 35). The shades of pink represent the different scenarios of the immune status of the first infected duck (light pink: nonimmune; pink: immune; dark pink: randomly selected with a probability of 0.2 to be nonimmune and 0.8 to be immune). Each probability was calculated based on 500 stochastic simulations of the model.

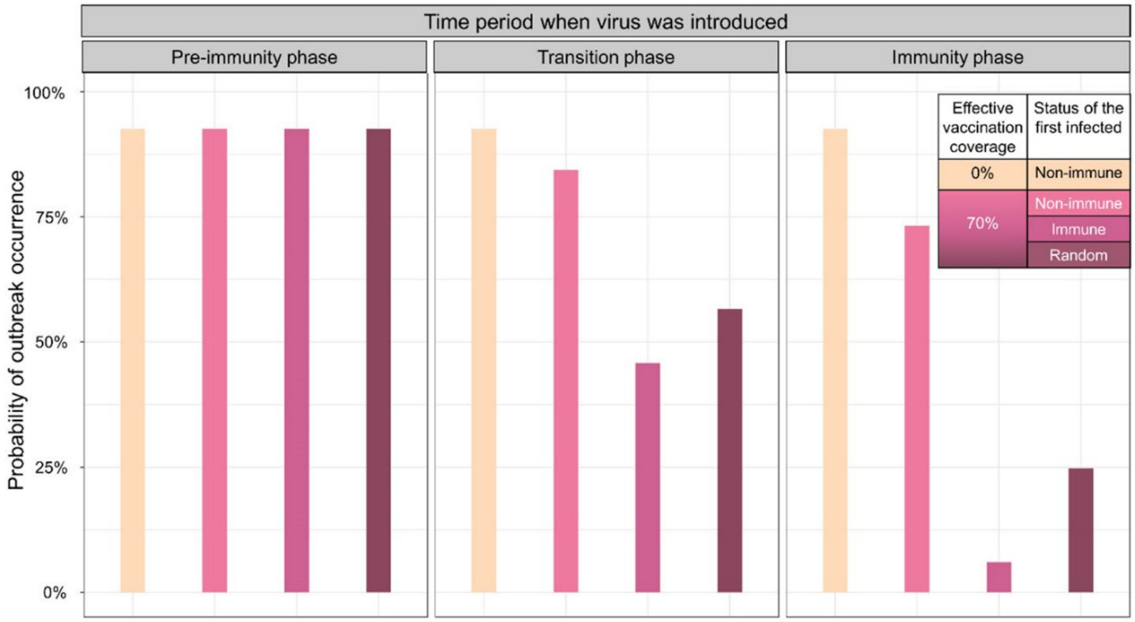

Appendix Figure 2. Probability of outbreak occurrence in unvaccinated (beige bars) and preventively vaccinated duck flocks (pink bars), for different timings of virus introduction. Effective vaccination coverage in vaccinated flocks was assumed to be 70%. Outbreak was defined as a simulation where at least five ducks became infected following the first infected duck. Left panel: the virus was introduced into the flock when ducks were not yet immune (i.e., before day 28); middle panel: the virus was introduced during the transition phase (i.e., between day 28 and day 35); right panel: the virus was introduced once immunity was fully reached (i.e., after day 35). The shades of pink represent the different scenarios of the immune status of the first infected duck (light pink: nonimmune; pink: immune; dark pink: randomly selected with a probability of 0.3 to be nonimmune and 0.7 to be immune). Each probability was calculated based on 500 stochastic simulations of the model.

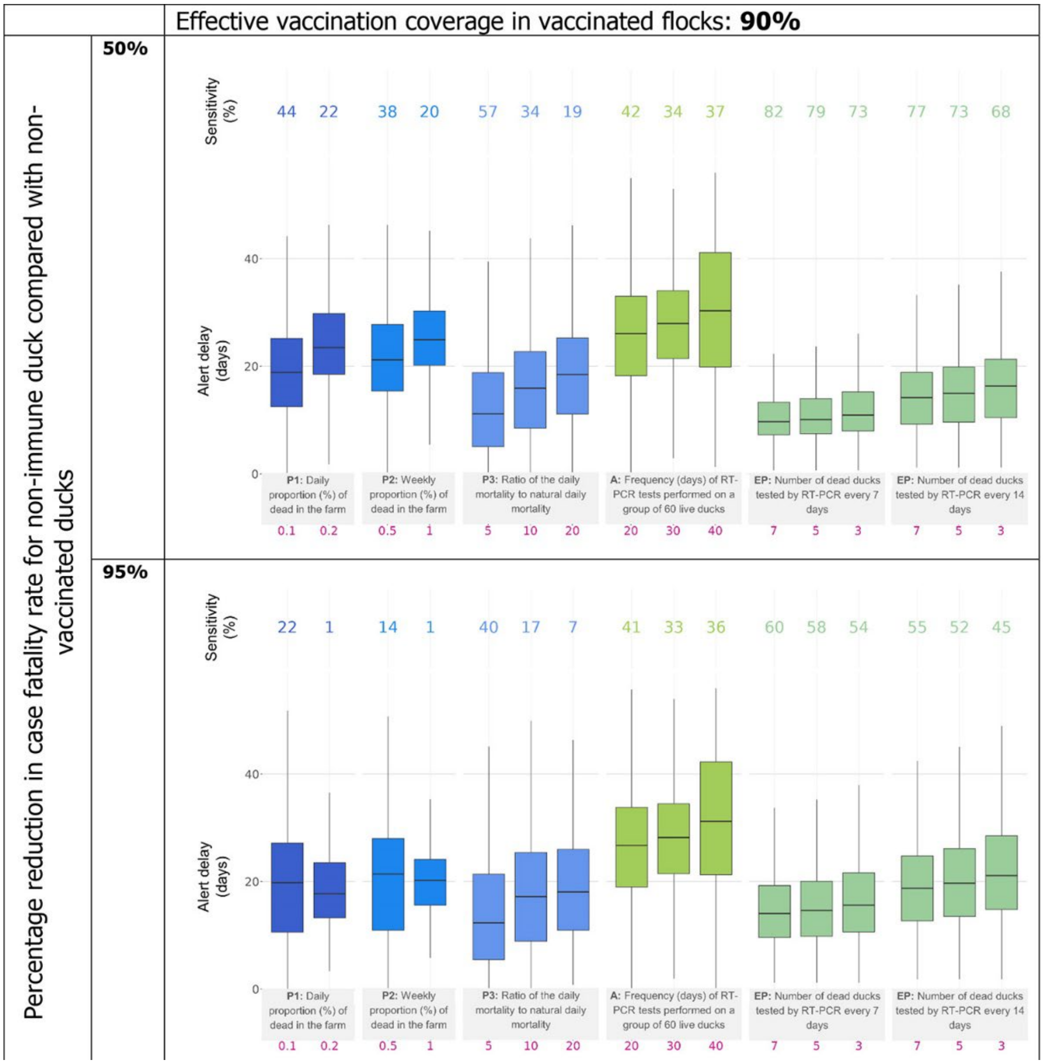

Appendix Figure 3. Comparison of the sensitivity and alert delay of different surveillance strategies in preventively vaccinated farms, according to different values of case fatality rate for nonimmune vaccinated ducks. Effective vaccination coverage in vaccinated flocks was assumed to be 90%. For each of the surveillance strategies, two or three scenarios were tested by varying the value of x (in pink). For passive surveillance strategies P1, P2 and P3, x referred to mortality thresholds. For active surveillance A, x referred to the frequency with which 60 live ducks were taken from the farm. For enhanced passive surveillance EP, x referred to the number of dead ducks sampled each time. For each of these scenarios, the sensitivity and alert delay were compared. Sensitivity (first row of each graph) was the percentage of outbreaks out of 5,000 that triggered an alert. Alert delay (second row of each graph) was the distribution of the number of days between the virus introduction and the alert, out of 5,000 outbreaks. Here we compared the effectiveness of these different surveillance strategies according to different percentage of reduction of the assumed case fatality rate for the vaccinated but nonimmune population, compared with the observed rates in unvaccinated ducks.

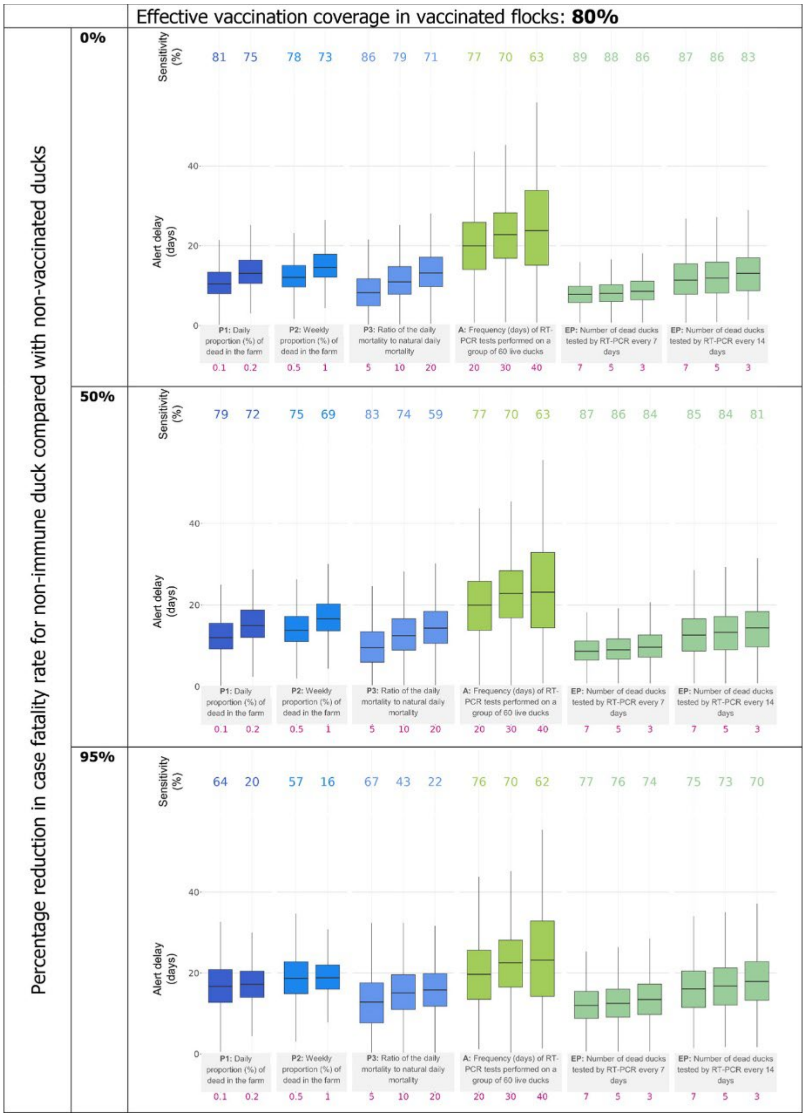

Appendix Figure 4. Comparison of the sensitivity and alert delay of different surveillance strategies in preventively vaccinated farms, according to different values of case fatality rate of nonimmune vaccinated ducks. Effective vaccination coverage in vaccinated flocks was assumed to be 80%. For each of the surveillance strategies, two or three scenarios were tested by varying the value of x (in pink). For passive surveillance strategies P1, P2 and P3, x referred to mortality thresholds. For active surveillance A, x referred to the frequency with which 60 live ducks were taken from the farm. For enhanced passive surveillance EP, x referred to the number of dead ducks sampled each time. For each of these scenarios, the sensitivity and alert delay were compared. Sensitivity (first row of each graph) was the percentage of outbreaks out of 5,000 that triggered an alert. Alert delay (second row of each graph) was the distribution of the number of days between the virus introduction and the alert, out of 5,000 outbreaks. Here we compared the effectiveness of these different surveillance strategies according to different percentage of reduction of the assumed case fatality rate for the vaccinated but nonimmune population, compared with the observed rates in unvaccinated ducks.

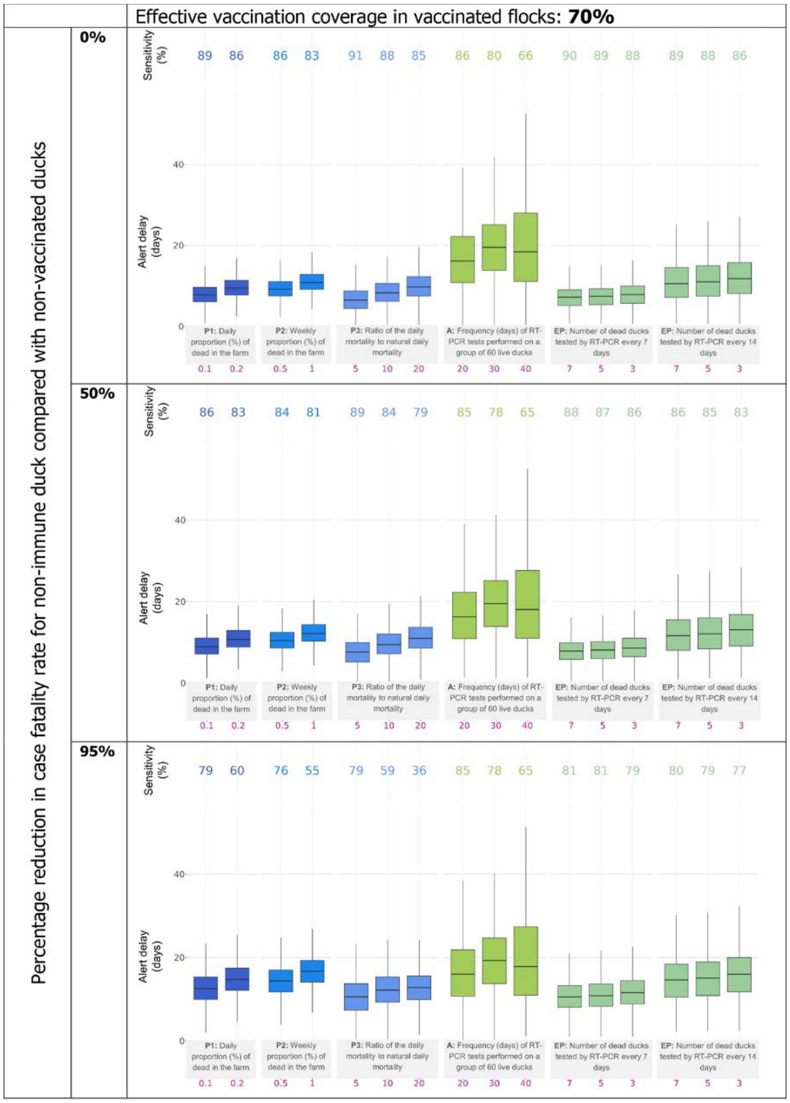

Appendix Figure 5. Comparison of the sensitivity and alert delay of different surveillance strategies in preventively vaccinated farms, according to different values of case fatality rate of nonimmune vaccinated ducks. Effective vaccination coverage in vaccinated flocks was assumed to be 70%. For each of the surveillance strategies, two or three scenarios were tested by varying the value of x (in pink). For passive surveillance strategies P1, P2 and P3, x referred to mortality thresholds. For active surveillance A, x referred to the frequency with which 60 live ducks were taken from the farm. For enhanced passive surveillance EP, x referred to the number of dead ducks sampled each time. For each of these scenarios, the sensitivity and alert delay were compared. Sensitivity (first row of each graph) was the percentage of outbreaks out of 5,000 that triggered an alert. Alert delay (second row of each graph) was the distribution of the number of days between the virus introduction and the alert, out of 5,000 outbreaks. Here we compared the effectiveness of these different surveillance strategies according to different percentage of reduction of the assumed case fatality rate for the vaccinated but nonimmune population, compared with the observed rates in unvaccinated ducks.
